# Design, Synthesis and Biological Evaluation of Benzo[D]Thiazole with Dimethylpyrazol Derivatives

**DOI:** 10.31557/APJCP.2019.20.11.3487

**Published:** 2019

**Authors:** Dachuan Liu, Xiu Cheng, Ying Wang

**Affiliations:** 1 *School of Pharmacy, *; 2 *School of Public Foundation, Bengbu Medical College, Bengbu, China. *

**Keywords:** Benzothiazole, dimethylpyrazol, maximal electroshock, MTT assay, GABAergic

## Abstract

A series of new benzothiazole derivatives containing dimethylpyrazole were synthesized and evaluated for their anticonvulsant activity, neurotoxicity and cytotoxicity by using the maximal electroshock (MES), rotarod neurotoxicity (TOX) and MTT colorimetric assay. Among the compounds studied, four compounds (**6a, 6b, 6g** and **6m**) showed better anticonvulsant than the others at 300 mg/kg and they also showed anticonvulsant activity at the dose of 100 mg/kg. All the synthetic compounds showed lower neurotoxicity and little cytotoxicity, so that the compounds, which with better activities, also had higher protective index. In particular, the compound **6g**, 2-(3,5-dimethyl-1H-pyrazol-1-yl)-6-((2-fluorobenzyl)oxy)benzo[d]thiazole showed better activity with an ED_50_ value of 160.4 mg/kg and higher protective index (PI) values of 2.74 in the MES test than the standard drugs sodium valproate, which used as positive controls in this study. After that the compound **6g** demonstrated antagonistic activity against seizures induced by pentylenetetrazol, which proved **6g** maybe exert activity through effecting GABAergic neurotransmission.

## Introduction

Epilepsy is a chronic noncommunicable disease of the brain that affects people of all ages, which affects approximately 50 million people worldwide (Detail, 2019). Currently, 40% of patients in high-income countries and more than 70% of patients in developing countries do not get the treatment they need, because of the high expense or low availability of the appropriate drugs (Van-Nieuwenhuyse et al., 2015; Editorial, 2015). Therefore, there is a pressing need to develop more effective antiepileptic drugs (AEDs) endowed with an improved safety profile.

**Figure 1:**
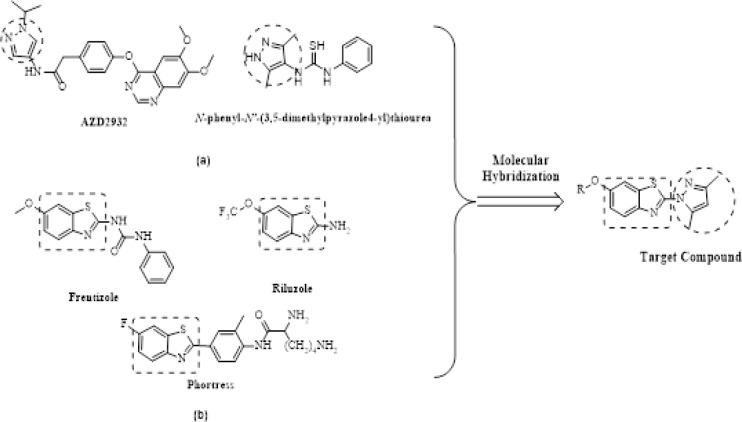
Rational Design of the Target Compounds. (a) Examples of pyrazole and 3,5-dimethylpyrazol derivatives with biological activity. (b) Representative examples of benzothiazole derivatives

On the basis of related materials, pyrazoles, especially 3, 5-dimethylpyrazol, occupy a distinct niche in heterocyclic chemistry and represent a key motif in medicinal chemistry because of their capability to exhibit an array of properties and bioactivities, including fungicidal (Wang et al., 2012), anticancer (Ganga-Reddy et al., 2016), anti-inflammatory (Hu et al., 2018), anticonvulsant (Kaymakcioğlu et al., 2003), and nitrification inhibitor (Şahan et al., 2017) activities. Furthermore, we also demonstrated that the benzothiazole nucleus is a unique scaffold for further molecular exploration to synthesize novel compounds. A literature survey revealed that benzothiazole analogs are associated with diverse pharmacological effects (Agarwal et al., 2014; Singh et al., 2013; Stone et al., 2015; Ma et al., 2015), including anticonvulsant activity (Ugale et al., 2012; Siddiqui et al., 2007). For these reasons and in continuation to our efforts directed toward the synthesis of new heterocyclic compounds with anticonvulsant biological activities, in this study, we combined both biological components 3, 5-dimethylpyrazol and benzothiazole, to obtain a series of 2-(3,5-dimethyl-1H-pyrazol-1-yl)-6-alkoxybenzo[d]thiazole or 2-(3,5-dimethyl-1H-pyrazol-1-yl)-6-benzylbenzo[d]thiazole (The design thought and the structure of target compounds were shown in Figure 1). Their anticonvulsant activities were evaluated using the maximal electroshock (MES) test and subcutaneous pentylenetetrazole (scPTZ), their neurotoxicity (TOX) was evaluated with the rotarod test in mice.

*Experimental Procedures*

*General Information*

Melting points were determined in open capillary tubes and were uncorrected. IR spectra were recorded (in KBr) on IR Prestige-21. ^1^H-NMR and ^13^C-NMR spectra were measured on an AV-300 (Bruker, Switzerland), and all chemical shifts were given in ppm relative to tetramethylsilane. Mass spectra were measured on an AXIMA CFR Plus MALDI-TOF (Shimadzu, Japan). Elemental analyses were performed on a 204Q CHN (Perkin Elmer, USA).The chemicals were purchased from Aldrich Chemical Corporation. All other chemicals were of analytical grade.

*Synthesis Method*

*Synthesis of 6-methoxybenzo[d]thiazol-2-amine (* ***2*** *)*

A mixture of 4-methoxyaniline (50 mmol, 6.15g) and 19.0 g ammonium sulfocyanate (5 e.q., 250mmol) in 50 mL acetic acid was stirred for 30 min at room temperature. Then, 3.0 mL acetic acid solution, containing 0.055 mol bromine, was dropwise added with stirring and cooling in ice bath. And after that, the reaction continued at room temperature for 5 h. The mixture was added into 500 mL water and adjusted with ammonia solution to pH 9, the product precipitate out. Then it was filtered to obtain a yellow-white solid (compound **2**) with 69% yield. 

*Synthesis of 2-hydrazinyl-6-methoxybenzo[d]thiazole (* ***3*** *)*

20mmol compound **2** (3.60 g) and 0.6 mL 98% H_2_SO_4_ solution (water solutions) in 20 mL of glycol was refluxed for 0.5 h at 80^o^C. Then 10ml of hydrazine hydrate was added into the mixture and heated at 140^o^C for 5 h. After cooling to room temperature, the mixture was added into 50 mL of ice-water. The precipitate formed was filtered and washed with water to obtain a light green needle-like solid. The yield was 60%.

*Synthesis of 2-(3, 5-dimethyl-1H-pyrazol-1-yl)-6-methoxybenzo[d]thiazole (* ***4*** *)*

A mixture of compound **3** (10 mmol) and equimolar amounts of acetylacetone were added into a flask with 30 mL dioxane as solvent and refluxed reacted for 1 h at 100^o^C. After removing the solvent under reduced pressure, the crude product was obtained. Then crude product was purified by silica gel column chromatography with CH_2_Cl_2_ to get compound **4** (72.3% yield) as a white solid. Melting point and spectral data of compound **4** was given: M.p. 124-126^o^C. ^1^H NMR (300 MHz, CDCl_3_) δ 7.76 (d, *J* = 8.9 Hz, 1H, Ar-H), 7.31 (d, *J* = 2.3 Hz, 1H, Ar-H), 7.05 (d, *J* = 8.9 Hz, 1H, Ar-H), 6.04 (s, 1H, pyrazole-H), 3.90 (s, 3H, -OCH_3_), 2.77 (s, 3H, pyrazole-CH_3_), 2.32 (s, 3H, pyrazole-CH_3_). ^13^C NMR (75 MHz, CDCl_3_) δ 159.17 (s), 157.11 (s), 151.80 (s), 145.81 (s), 142.17 (s), 134.13 (s), 122.83 (s), 114.85 (s), 109.97 (s), 104.30 (s), 55.76 (s), 13.82 (s), 13.62 (s). IR (KBr) cm^−1^: 1604.77 (C=N), 1577.77 (C=C). MS-EI m/z 260 (M + H^+^). Anal. Calcd. for C_13_H_13_N_3_OS: C, 60.21; H, 5.05; N, 16.20. Found: C, 60.32; H, 5.25; N, 16.07.

*Synthesis of 2-(3, 5-dimethyl-1H-pyrazol-1-yl)benzo[d]thiazol-6-ol (* ***5*** *)*

2-(3,5-dimethyl-1H-pyrazol-1-yl)-6-methoxybenzo[d]thiazole (compound **4**) (1.5 g, 5.8 mmol) was dissolved in 50 mL dichloromethane. BBr_3_ (29.0 mmol) was added dropwise to the solution and then the mixture was stirred at room temperature. After 4 h the mixture was added slowly 20 mL ice cold water and continue to stir for 0.5 h. The resulting white precipitate was obtained by filtration with 38.8% yield. Melting point and spectral data of compound 5 was given: M.p. 186-188^o^C. ^1^H NMR (300 MHz, CDCl_3_) δ 7.73 (d, *J* = 8.7 Hz, 1H), 7.28 (s, 1H), 6.96 (d, *J* = 8.7 Hz, 1H), 6.05 (s, 1H), 5.30 (s, 1H), 2.77 (s, 3H), 2.32 (s, 3H). ^13^C NMR (75 MHz, CDCl3) δ 160.53 (s), 157.77 (s), 153.05 (s), 146.74 (s), 146.19 (s), 134.41 (s), 123.03 (s), 115.12 (s), 110.02 (s), 107.00 (s), 90.41 (s), 13.71 (s), 13.56 (s). IR (KBr) cm^−1^: 1602.81 (C=N), 1579.77 (C=C). MS-EI m/z 246 (M + H^+^).

*General procedure for the synthesis of 2-(3, 5-dimethyl-1H-pyrazol-1-yl)- 6-alkoxybenzo[d]thiazole (* ***6a-6o*** *)*

Compound **5** (3.0 mmol), potassium carbonate (3.6 mmol) were dissolved in 50 mL acetonitrile and refluxed for 30 min, then appropriate alkyl bromide or benzyl chloride derivatives (3.3 mmol) and a catalytic amount of benzyltriethylamine chloride (TEBA) were added into the mixture. The reaction mixture was heated at reflux temperature for 12-48 h. After removing the solvent under reduced pressure, 50 mL water was poured into the mixture and stirred for 0.5 h to remove the excess K_2_CO_3_. The remaining precipitate was filtered to obtain a white solid. The yield, melting point and spectral data of each compound were given below.

*6-butoxy-2-(3,5-dimethyl-1H-pyrazol-1-yl)benzo[d]thiazole* **6a**

yield: 76.52%. m.p. 92-94 ^o^C. ^1^H NMR (300 MHz, CDCl_3_) δ 7.75 (d, *J* = 8.9 Hz, 1H, Ar-H), 7.30 (s, 1H, Ar-H), 7.04 (d, *J* = 8.7 Hz, 1H, Ar-H), 6.04 (s, 1H, pyrazole-H), 4.04 (t, *J* = 6.4 Hz, 2H, -OCH_2_-), 2.77 (s, 3H, pyrazole-CH_3_), 2.32 (s, 3H, pyrazole-CH_3_), 1.94 – 1.74 (m, 2H, -CH_2_-), 1.59 – 1.45 (m, 2H, -CH_2_-), 1.02 (t, *J* = 7.1 Hz, 3H, -CH_3_). ^13^C NMR (75 MHz, CDCl_3_) δ 156.69 (s), 151.77 (s), 145.68 (s), 142.16 (s), 134.10 (s), 122.78 (s), 115.31 (s), 109.93 (s), 105.04 (s), 68.34 (s), 31.34 (s), 19.26 (s), 13.86 (s), 13.82 (s), 13.62 (s). IR (KBr) cm^−1^: 1604.77 (C=N), 1577.77 (C=C). MS-EI m/z 302 (M + H+). Anal. Calcd. for C_16_H_19_N_3_OS: C, 63.76; H, 6.35; N, 13.94. Found: C, 63.91; H, 6.43; N, 13.85.

*2-(3,5-dimethyl-1H-pyrazol-1-yl)-6-(pentyloxy)benzo[d]thiazole* ***6b***

yield: 61.28%. m.p. 89-91^o^C. 1H NMR (300 MHz, CDCl_3_) δ 7.75 (d, *J* = 8.9 Hz, 1H, Ar-H), 7.30 (d, *J* = 2.5 Hz, 1H, Ar-H), 7.04 (d, *J* = 8.9 Hz, 1H, Ar-H), 6.04 (s, 1H, pyrazole-H), 4.03 (t, *J* = 6.6 Hz, 2H, -OCH_2_-), 2.77 (s, 3H, pyrazole-CH_3_), 2.32 (s, 3H, pyrazole-CH_3_), 1.83 (dd, *J* = 14.4, 6.8 Hz, 2H, -CH_2_-), 1.54 – 1.33 (m, 4H, -(CH_2_)_2_-), 0.96 (t, *J* = 7.1 Hz, 3H, -CH3). ^13^C NMR (75 MHz, CDCl_3_) δ 158.99 (s), 156.63 (s), 151.70 (s), 145.61 (s), 142.09 (s), 134.04 (s), 122.72 (s), 115.24 (s), 109.87 (s), 104.97 (s), 68.58 (s), 28.93 (s), 28.15 (s), 22.41 (s), 13.97 (s), 13.75 (s), 13.55 (s). IR (KBr) cm^−1^: 1604.77 (C=N), 1577.77 (C=C). MS-EI m/z 316 (M + H^+^). Anal. Calcd. for C_17_H_21_N_3_OS: C, 64.73; H, 6.71; N, 13.32. Found: C, 64.82; H, 6.85; N, 13.18.

*2-(3,5-dimethyl-1H-pyrazol-1-yl)-6-(hexyloxy)benzo[d]thiazole* ***6c***

yield: 67.71%. m.p. 78-80^o^C. ^1^H NMR (300 MHz, CDCl3) δ 7.75 (d, *J* = 9.0 Hz, 1H, Ar-H), 7.30 (d, *J* = 2.7 Hz, 1H, Ar-H), 7.04 (d, *J* = 8.8 Hz, 1H, Ar-H), 6.04 (s, 1H, pyrazole-H), 4.03 (t, *J* = 6.5 Hz, 2H, -OCH_2_-), 2.77 (s, 3H, pyrazole-CH_3_), 2.32 (s, 3H, pyrazole-CH_3_), 2.00 – 1.74 (m, 2H, -CH_2_-), 1.82-1.40 (m, 2H, -CH_2_-), 1.46-1.19 (m, 4H, -(CH_2_)_2_-), 0.94 (t, *J* = 7.1 Hz, 3H, -CH_3_). ^13^C NMR (75 MHz, CDCl_3_) δ 159.00 (s), 156.63 (s), 151.70 (s), 145.62 (s), 142.08 (s), 134.04 (s), 122.72 (s), 115.24 (s), 109.87 (s), 104.97 (s), 68.59 (s), 31.53 (s), 29.20 (s), 25.68 (s), 22.55 (s), 13.98 (s), 13.75 (s), 13.56 (s). IR (KBr) cm^−1^: 1608.63(C=N), 1577.77 (C=C). MS-EI m/z 330 (M + H^+^). Anal. Calcd. for C_18_H_23_N_3_OS: C, 65.62; H, 7.04; N, 12.75. Found: C, 65.78; H, 7.15; N, 12.66.

*2-(3,5-dimethyl-1H-pyrazol-1-yl)-6-(heptyloxy)benzo[d]thiazole* ***6d***

yield*: *70.32%. m.p. 85-87^o^C. ^1^H NMR (300 MHz, CDCl_3_) δ 7.75 (d, *J *= 8.9 Hz, 1H, Ar-H), 7.30 (d, *J* = 2.5 Hz, 1H, Ar-H), 7.04 (dd, *J* = 8.9, 2.5 Hz, 1H, Ar-H), 6.04 (s, 1H, pyrazole-H), 4.03 (t, *J* = 6.5 Hz, 2H, -OCH_2_-), 2.77 (s, 3H, pyrazole-CH_3_), 2.32 (s, 3H, pyrazole-CH_3_), 1.95 – 1.74 (m, 2H, -CH_2_-), 1.55 – 1.27 (m, 8H, -(CH_2_)_4_-), 0.92 (t,* J *= 6.6 Hz, 3H, -CH_3_). ^13^C NMR (75 MHz, CDCl_3_) δ 159.00 (s), 156.63 (s), 151.69 (s), 145.61 (s), 142.07 (s), 134.03 (s), 122.72 (s), 115.24 (s), 109.86 (s), 104.96 (s), 68.59 (s), 31.73 (s), 29.13 (d, *J* = 17.1 Hz), 25.96 (s), 22.56 (s), 14.04 (s), 14.04 (s), 13.75 (s), 13.56 (s). IR (KBr) cm^−1^: 1608.63 (C=N), 1577.77 (C=C). MS-EI m/z 344 (M + H^+^). Anal. Calcd. for C_19_H_25_N_3_OS: C, 66.44; H, 7.34; N, 12.23. Found: C, 66.61; H, 7.51; N, 12.10.

*2-(3,5-dimethyl-1H-pyrazol-1-yl)-6-(octyloxy)benzo[d]thiazole* ***6e***

yield: 62.78%. m.p. 80-82^o^C. 1H NMR (300 MHz, CDCl_3_) δ 7.75 (d, *J* = 8.9 Hz, 1H, Ar-H), 7.30 (d, *J* = 2.5 Hz, 1H, Ar-H), 7.04 (dd, *J* = 8.9, 2.5 Hz, 1H, Ar-H), 6.04 (s, 1H, pyrazole-H), 4.03 (t,* J* = 6.5 Hz, 2H, -OCH_2_-), 2.77 (s, 3H, pyrazole-CH_3_), 2.32 (s, 3H, pyrazole-CH_3_), 1.96 – 1.73 (m, 2H, -CH_2_-), 1.60- 1.12 (m, 10H, -(CH_2_)_5_-), 0.90 (t, *J* = 7.0 Hz, 3H, -CH_3_). ^13^C NMR (75 MHz, CDCl_3_) δ 159.00 (s), 156.63 (s), 151.70 (s), 145.61 (s), 142.08 (s), 134.03 (s), 122.72 (s), 115.24 (s), 109.86 (s), 104.97 (s), 68.60 (s), 31.76 (s), 29.24 (s), 26.00 (s), 22.61 (s), 14.05 (s), 14.05 (s), 13.75 (s), 13.56 (s). IR (KBr) cm^−1^: 1608.63 (C=N), 1577.77 (C=C). MS-EI m/z 358 (M + H^+^). Anal. Calcd. for C_20_H_27_N_3_OS: C, 67.19; H, 7.61; N, 11.75. Found: C, 67.30; H, 7.72; N, 11.68.

*6-(benzyloxy)-2-(3,5-dimethyl-1H-pyrazol-1-yl)benzo[d]thiazole* ***6f***

 yield: 56.18%. m.p. 117-119^ o^C. ^1^H NMR (300 MHz, CDCl3) δ 7.77 (d, *J* = 8.9 Hz, 1H, Ar-H), 7.58 – 7.34 (m, 6H, Ar-H), 7.12 (dd, *J* = 8.9, 2.5 Hz, 1H, Ar-H), 6.05 (s, 1H, pyrazole-H), 5.15 (s, 2H, -OCH_2_-), 2.77 (s, 3H, pyrazole-CH_3_), 2.33 (s, 3H, pyrazole-CH_3_). ^13^C NMR (75 MHz, CDCl_3_) δ 159.27 (s), 156.19 (s), 151.77 (s), 145.97 (s), 142.11 (s), 136.64 (s), 134.04 (s), 128.55 (s), 128.00 (s), 127.44 (s), 122.80 (s), 115.45 (s), 109.93 (s), 105.57 (s), 70.57 (s), 13.76 (s), 13.56 (s). IR (KBr) cm^−1^: 1604.77 (C=N), 1579.70 (C=C). MS-EI m/z 336 (M + H^+^). Anal. Calcd. for C_19_H_17_N_3_OS: C, 68.04; H, 5.11; N, 12.53. Found: C, 68.19; H, 5.02; N, 12.57.

*2-(3,5-dimethyl-1H-pyrazol-1-yl)-6-((2-fluorobenzyl)oxy)benzo[d]thiazole* ***6g***

yield: 61.18%. m.p. 126-128^o^C. ^1^H NMR (300 MHz, CDCl_3_) δ 7.78 (d, *J* = 8.9 Hz, 1H, Ar-H), 7.56 (t, *J* = 7.3 Hz, 1H, Ar-H), 7.42 (d, *J* = 2.4 Hz, 1H, Ar-H), 7.36 (d, *J *= 5.9 Hz, 1H, Ar-H), 7.22 (d, *J* = 7.5 Hz, 1H, Ar-H), 7.18 – 7.05 (m, 2H, Ar-H), 6.05 (s, 1H, pyrazole-H), 5.22 (s, 2H, -OCH_2_-), 2.78 (s, 3H, pyrazole-CH_3_), 2.33 (s, 3H, pyrazole-CH_3_). ^13^C NMR (75 MHz, CDCl_3_) δ 162.02 (s), 159.37 (s), 158.74 (s), 155.93 (s), 151.79 (s), 146.12 (s), 142.12 (s), 134.05 (s), 129.68 (dd, *J* = 7.9, 6.1 Hz), 124.20 (d, *J* = 3.5 Hz), 122.81 (s), 115.31 (t, *J* = 10.6 Hz), 109.94 (s), 105.66 (s), 64.34 (s), 13.74 (s), 13.54 (s). IR (KBr) cm^−1^: 1604.77 (C=N); 1577.97 (C=C). MS-EI m/z 354 (M + H^+^). Anal. Calcd. for C_19_H_16_FN_3_OS: C, 64.57; H, 4.56; N, 11.89. Found: C, 64.69; H, 4.62; N, 11.67.

*2-(3,5-dimethyl-1H-pyrazol-1-yl)-6-((3-fluorobenzyl)oxy)benzo[d]thiazole* ***6h***

 yield: 63.36%. m.p. 101-103^o^C. ^1^H NMR (300 MHz, CDCl_3_) δ 7.78 (d, *J* = 8.9 Hz, 1H, Ar-H), 7.38 (s, 2H, Ar-H), 7.28 – 7.15 (m, 2H, Ar-H), 7.11 (d, *J* = 8.9 Hz, 1H, Ar-H), 7.05 (s, 1H, Ar-H), 6.05 (s, 1H, pyrazole-H), 5.14 (s, 2H, -OCH_2_-), 2.77 (s, 3H, pyrazole-CH_3_), 2.32 (s, 3H, pyrazole-CH_3_). ^13^C NMR (75 MHz, CDCl_3_) δ 164.54 (s), 161.27 (s), 159.39 (s), 155.86 (s), 151.80 (s), 146.14 (s), 142.12 (s), 139.26 (d, *J *= 7.3 Hz), 134.07 (s), 130.08 (dd, *J* = 8.7, 3.9 Hz), 123.04 – 122.13 (m), 115.33 (s), 115.13 (d, *J* = 28.9 Hz), 114.66 (s), 114.27 (s), 113.98 (s), 109.94 (s), 105.65 (s), 69.72 (s), 13.71 (s), 13.51 (s). IR (KBr) cm^−1^: 1604.77 (C=N), 1597.77 (C=C). MS-EI m/z 354 (M + H^+^). Anal. Calcd. for C_19_H_16_FN_3_OS: C, 64.57; H, 4.56; N, 11.89. Found: C, 64.73; H, 4.60; N, 11.71.

*2-(3,5-dimethyl-1H-pyrazol-1-yl)-6-((4-fluorobenzyl)oxy)benzo[d]thiazole* ***6i***

 yield: 72.10%. m.p. 97-99^o^C. ^1^H NMR (300 MHz, CDCl3) δ 7.77 (d, *J* = 8.9 Hz, 1H, Ar-H), 7.45 (dd, *J* = 8.4, 5.5 Hz, 2H, Ar-H), 7.38 (d, *J* = 2.5 Hz, 1H, Ar-H), 7.22 – 7.04 (m, 3H, Ar-H), 6.05 (s, 1H, pyrazole-H), 5.10 (s, 2H, -OCH_2_-), 2.77 (s, 3H, pyrazole-CH_3_), 2.32 (s, 3H, pyrazole-CH_3_). ^13^C NMR (75 MHz, CDCl_3_) δ 164.09 (s), 160.82 (s), 159.34 (s), 155.99 (s), 151.79 (s), 146.06 (s), 142.11 (s), 134.05 (s), 132.40 (s), 129.27 (d, *J* = 8.1 Hz), 122.81 (s), 115.47 (d, *J* = 14.5 Hz), 109.95 (s), 105.59 (s), 69.89 (s), 13.72 (s), 13.52 (s). IR (KBr) cm^−1^: 1604.77 (C=N); 1573.91 (C=C). MS-EI m/z 354 (M + H^+^). Anal. Calcd. for C_19_H_16_FN_3_OS: C, 64.57; H, 4.56; N, 11.89. Found: C, 64.77; H, 4.59; N, 11.66.

*6-((2-chlorobenzyl)oxy)-2-(3,5-dimethyl-1H-pyrazol-1-yl)benzo[d]thiazole* ***6j***

yield: 52.52%. m.p. 108-110 ^o^C. ^1^H NMR (300 MHz, CDCl_3_) δ 7.79 (dd, *J* = 8.8, 2.6 Hz, 1H, Ar-H), 7.67 – 7.58 (m, 1H, Ar-H), 7.49 – 7.39 (m, 2H, Ar-H), 7.32 (dd, *J* = 6.3, 3.1 Hz, 2H, Ar-H), 7.21 – 7.10 (m, 1H, Ar-H), 6.05 (s, 1H, pyrazole-H), 5.25 (s, 2H, -OCH_2_-), 2.77 (s, 3H, pyrazole-CH_3_), 2.33 (s, 3H, pyrazole-CH_3_). ^13^C NMR (75 MHz, CDCl_3_) δ 159.41 (s), 155.89 (s), 151.78 (s), 146.17 (s), 142.12 (s), 134.24 (d, *J *= 22.1 Hz), 132.56 (s), 129.32 (s), 129.13 – 129.04 (m), 128.85 (d, *J* = 21.1 Hz), 126.89 (s), 122.83 (s), 115.30 (s), 109.93 (s), 105.77 (s), 67.74 (s), 13.73 (s), 13.53 (s). IR (KBr) cm^−1^: 1602.82 (C=N), 1573.91 (C=C). MS-EI m/z 370 (M + H^+^). Anal. Calcd. for C_19_H_16_ClN_3_OS: C, 61.70; H, 4.36; N, 11.36. Found: C, 61.81; H, 4.26; N, 11.54. 

*6-((3-chlorobenzyl)oxy)-2-(3,5-dimethyl-1H-pyrazol-1-yl)benzo[d]thiazole* ***6k***

yield: 78.96%. m.p. 112-114 ^o^C. ^1^H NMR (300 MHz, CDCl_3_) δ 7.86 – 7.73 (m, 1H, Ar-H), 7.56 – 7.47 (m, 1H, Ar-H), 7.46 – 7.30 (m, 4H, Ar-H), 7.18 – 7.05 (m, 1H, Ar-H), 6.06 (s, 1H, pyrazole-H), 5.13 (s, 2H, -OCH_2_-), 2.78 (s, 3H, pyrazole-CH_3_), 2.33 (s, 3H, pyrazole-CH_3_). 13C NMR (75 MHz, CDCl_3_) δ 159.52 (s), 155.95 (s), 151.92 (s), 146.27 (s), 142.24 (s), 138.83 (s), 134.57 (s), 134.18 (s), 129.91 (s), 128.20 (s), 127.45 (s), 125.38 (s), 122.96 (s), 115.42 (s), 110.08 (s), 105.75 (s), 69.80 (s), 13.86 (s), 13.66 (s). IR (KBr) cm^−1^: 1602.85 (C=N), 1573.91(C=C). MS-EI m/z 370 (M + H^+^). Anal. Calcd. for C_19_H_16_ClN_3_OS: C, 61.70; H, 4.36; N, 11.36. Found: C, 61.85; H, 4.19; N, 11.41.

*6-((4-chlorobenzyl)oxy)-2-(3,5-dimethyl-1H-pyrazol-1-yl)benzo[d]thiazole* ***6l***

yield: 70.62%. m.p. 110-112 ^o^C. ^1^H NMR (300 MHz, CDCl3) δ 7.77 (d, *J* = 8.9 Hz, 1H, Ar-H), 7.42 (d, *J* = 9.8 Hz, 4H, Ar-H), 7.37 (d, *J* = 2.5 Hz, 1H, Ar-H), 7.10 (dd, *J* = 8.9, 2.5 Hz, 1H, Ar-H), 6.05 (s, 1H, pyrazole-H), 5.11 (s, 2H, -OCH_2_-), 2.77 (s, 3H, pyrazole-CH_3_), 2.32 (s, 3H, pyrazole-CH_3_). ^13^C NMR (75 MHz, CDCl_3_) δ 159.49 (s), 156.02 (s), 151.93 (s), 146.22 (s), 142.24 (s), 135.25 (s), 134.17 (s), 133.88 (s), 128.81 (s), 122.95 (s), 115.45 (s), 110.09 (s), 105.73 (s), 69.87 (s), 13.86 (s), 13.66 (s). IR (KBr) cm^−1^: 1604.77 (C=N); 1573.91 (C=C). MS-EI m/z 370 (M + H^+^). Anal. Calcd. for C_19_H_16_ClN_3_OS: C, 61.70; H, 4.36; N, 11.36. Found: C, 61.79; H, 4.30; N, 11.45. 

*6-((2,6-dichlorobenzyl)oxy)-2-(3,5-dimethyl-1H-pyrazol-1-yl)benzo[d]thiazole* ***6m***

 yield: 52.80%. m.p. 128-130^o^C. ^1^H NMR (300 MHz, CDCl3) δ 7.79 (dd, *J* = 8.9, 1.9 Hz, 1H, Ar-H), 7.47 (d, *J *= 2.3 Hz, 1H, Ar-H), 7.44 – 7.24 (m, 3H, Ar-H), 7.21 – 7.11 (m, 1H, Ar-H), 6.05 (s, 1H, pyrazole-H), 5.36 (s, 2H, -OCH_2_-), 2.78 (s, 3H, pyrazole-CH_3_), 2.33 (s, 3H, pyrazole-CH_3_). ^13^C NMR (75 MHz, CDCl3) δ 159.59 (s), 156.37 (s), 151.87 (s), 146.47 (s), 142.25 (s), 137.07 (s), 134.14 (s), 132.06 (s), 130.77 (s), 130.46 (s), 128.50 (s), 122.91 (s), 115.79 (s), 115.38 (s), 110.01 (s), 106.21 (s), 66.13 (s), 13.76 (s), 13.59 (s). IR (KBr) cm^−1^: 1602.85 (C=N); 1573.91 (C=C). MS-EI m/z 404 (M + H^+^). Anal. Calcd. for C_19_H_15_C_l2_N_3_OS: C, 56.44; H, 3.74; N, 10.39. Found: C, 56.63; H, 3.90; N, 10.08. 

*2-(3,5-dimethyl-1H-pyrazol-1-yl)-6-((4-(trifluoromethyl)benzyl)oxy)benzo[d]thiazole* ***6n***

yield: 50.12%. m.p. 142-144^o^C. ^1^H NMR (300 MHz, CDCl3) δ 7.79 (d, *J* = 9.0 Hz, 1H, Ar-H), 7.76 (s, 1H, Ar-H), 7.67 (d, *J* = 7.3 Hz, 1H, Ar-H), 7.62 (s, 1H, Ar-H), 7.56 (d, *J* = 7.5 Hz, 1H, Ar-H), 7.40 (d, *J* = 2.4 Hz, 1H, Ar-H), 7.13 (dd, *J* = 9.0, 2.4 Hz, 1H, Ar-H), 6.05 (s, 1H, pyrazole-H), 5.19 (s, 2H, -OCH_2_-), 2.78 (s, 3H, pyrazole-CH_3_), 2.33 (s, 3H, pyrazole-CH_3_). ^13^C NMR (75 MHz, CDCl_3_) δ 159.57 (s), 155.89 (s), 151.97 (s), 146.34 (s), 142.26 (s), 137.79 (s), 134.19 (s), 131.23 (s), 130.61 (s), 129.11 (s), 127.40 (s), 124.87 (dd, *J* = 7.4, 3.7 Hz), 124.10 (dd, *J* = 7.4, 3.6 Hz), 122.99 (s), 115.38 (s), 110.10 (s), 105.74 (s), 69.82 (s), 13.84 (s), 13.64 (s). IR (KBr) cm^−1^: 1604.77 (C=N), 1573.91 (C=C). MS-EI m/z 404 (M + H^+^). Anal. Calcd. for C_20_H_16_F_3_N_3_OS: C, 59.55; H, 4.00; N, 10.42. Found: C, 59.72; H, 3.88; N, 10.58.

*2-(3,5-dimethyl-1H-pyrazol-1-yl)-6-((4-methylbenzyl)oxy)benzo[d]thiazole* ***6o***

yield: 72.52%. m.p. 110-112^o^C. ^1^H NMR (300 MHz, CDCl3) δ 7.76 (d, *J* = 8.9 Hz, 1H, Ar-H, Ar-H), 7.37 (d, *J *= 6.8 Hz, 3H, Ar-H), 7.23 (d, *J* = 8.0 Hz, 2H, Ar-H), 7.11 (dd, *J* = 8.8, 2.2 Hz, 1H, Ar-H), 6.05 (s, 1H, pyrazole-H), 5.10 (s, 2H, -OCH_2_-), 2.77 (s, 3H, pyrazole-CH_3_), 2.39 (s, 3H, Ar-CH_3_), 2.32 (s, 3H, pyrazole-CH_3_). ^13^C NMR (75 MHz, CDCl_3_) δ 159.34 (s), 156.40 (s), 151.85 (s), 146.04 (s), 142.22 (s), 137.89 (s), 134.15 (s), 133.72 (s), 129.34 (s), 127.70 (s), 122.89 (s), 115.61 (s), 110.03 (s), 105.67 (s), 70.64 (s), 21.26 (s), 13.87 (s), 13.68 (s). IR (KBr) cm^−1^: 1606.70 (C=N), 1577.77 (C=C). MS-EI m/z 350 (M + H^+^). Anal. Calcd. for C_20_H_19_N_3_OS: C, 68.74; H, 5.48; N, 12.02. Found: C, 68.85; H, 5.56; N, 11.93.

*Pharmacology*

All compounds were evaluated for anticonvulsant activities with KunMing mice in the 18-22 g weight range purchased from the Laboratory of Animal Research, Bengbu Medical College. The animals were maintained on a 12 h light/dark cycle and allowed free access to food and water, except during the time they were removed from their cages for testing. The experimental substances were dissolved in DMSO with 30% PEG 400 and administered intraperitoneally (i.p.) in a volume of 0.1 ml/20g body weight. The test method with reference to the Antiepileptic Drug Development (ADD) program (Krall et al., 1978; Porter et al., 1984).

*MES screening test*

Seizures were elicited with a 60-Hz alternating current of 50 mA intensity applied via corneal electrodes for 0.2 s. Protection against the spread of MES-induced seizures was defined as the abolition of the hind leg, and tonic maximal extension component of the seizure. The number of mice was three in each group in the screening test. The MES test was performed at thirty minutes after compound administration. 

*Neurotoxicity screening test*

The neurotoxicity of the compounds was measured in mice using the rota-rod test. Mice were tested on a knurled plastic rod (diameter, 3.2 cm) rotating at 6 rpm for 1 min, at 30 min after compound administration. The number of mice was three in each group. Neurotoxicity was measured by the inability of the animal to maintain equilibrium on the rod for at least 1 minute in each of the trials.

*Measurement of cell viability by MTT assay*

MDA-MB-231 cells (human breast cancer cells) were cultured in minimum essential medium (MEM, also obtained from Gibco) supplemented with 10% fetal bovine serum (FBS, Millipore, USA) and maintained at 37°C in a humidified incubator with 5% CO_2_. The cells were obtained from Shanghai Cell Bank, Chinese Academy of Sciences (CAS). The compounds were dissolved in dimethyl sulfoxide (DMSO, BIOSHARP, Hefei, China). The blank group was treated with DMSO only under identical conditions.

The 231 cells were seeded at 1 × 10^5^ cells/ml in 96-well plates containing 100 μl of DMEM medium with 10% FBS and incubated overnight. Compounds **4** and **6a-6o** was dissolved in DMSO, and the cells were pretreated with 20 μM concentration of the compounds for 24 h. Then the media was removed and cells were cultured with MTT solution (5 mg/ml) [3-(4, 5-dimethylthiazol-2-yl)-2, 5-diphenyltetrazolium bromide] (Sigma, St. Louis, MO, USA) for 4 h. The viable cells converted MTT to formazan, which generated a blue purple color after dissolving in 100 μl of DMSO. The absorbance at 570 nm was measured by Multiskan GO.

*ScPTZ seizures screening test*

At 1.5 h after the administration of the test compound, 85 mg/kg PTZ, which 100% of the mice showed tonic-clonic seizure and deaths, dissolved in saline was administered sc. The number of animals in each group tested was five. The animals placed in individual cages and observed for 1 h. The number of tonic-clonic seizure and deaths in mice were noted.

## Results

*Chemistry*

**Scheme 1:**
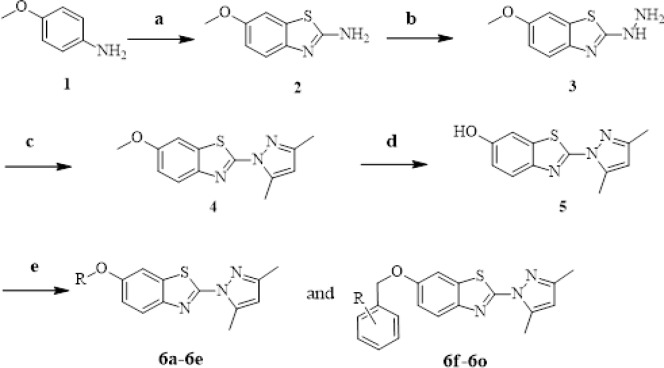
Synthetic Route of Target Compounds. Reagents and conditions: (a) Br_2_, NH_4_SCN, AcOH, 10 ºC to rt, 5 h; (b) NH_2_-NH_2_•H_2_O, Glycol, 98% H_2_SO_4_, 80 ^o^C, 0.5 h to 140 ^o^C, 5 h; (c) CH_3_COCH_2_COCH_3_, Dioxane, 100 ^o^C, 1h.(d) BBr_3_, CH_2_CCl_2_, 0 ^o^C to rt, 5 h; (e) RX, CH_3_CN, K_2_CO_3_, Reflux, 18-24 h

All the target compounds were synthesized according to Scheme 1. The starting material, 4-methoxyaniline (compound 1), was reacted with ammonium sulfocyanate by liquid bromine catalyzed to obtain 6-methoxy-1,3-benzothiazol-2-amine (compound **2**). Then compound 2 was treated with hydrazine hydrate in the presence of sulfuric acid (98% water solutions) to produce 2-hydrazinyl-6-methoxybenzo[d]thiazole (compound **3**) (Deng et al., 2010). 2-(3,5-dimethyl-1H-pyrazol-1-yl)-6-methoxybenzo[d]thiazole (compounds **4**) was synthesized from compound 3 and acetylacetone at 100 ºC in dioxane. Then compound **4** was reacted with hydrobromic acid (48% water solutions) to obtain compound **5** (Liu et al., 2014). Finally, compound **5** was converted to the target compounds **6a-6o** by reacting with appropriate alkanol and substituted phenol in acetonitrile under reflux conditions (Chen et al., 2004). Their chemical structures were characterized using ^1^H NMR, ^13^C NMR, IR and EI-MS. A detailed overview of their physical and analytical data has been provided in the experimental part. 

**Table 1:** Anticonvulsant Activities Screening (MES Test) in Mice at the Dose of 100 mg/kg, 300 mg/kg and Neurotoxicity Screening at the Dose of 300 mg/kg

Comp.	R	MES ^a^ (300mg/kg)		MES (100mg/kg)		TOX ^b^ (300mg/kg)	
		0.5 h	4 h	0.5 h	4 h	0.5 h	4 h
**4**	0	0/3 ^c^	0/3	- ^d^	-	0/3	0/3
**5**	H	0/3	0/3	-	-	0/3	0/3
**6a**	*n*-C_4_H_9_	2/3	0/3	1/3	0/3	0/3	0/3
**6b**	*n*-C_5_H_11_	3/3	0/3	1/3	0/3	0/3	0/3
**6c**	*n*-C_6_H_13_	1/3	0/3	-	-	0/3	0/3
**6d**	*n*-C_7_H_15_	0/3	0/3	-	-	0/3	0/3
**6e**	*n*-C_8_H_17_	0/3	0/3	-	-	0/3	0/3
**6f**	-CH_2_C_6_H_4_	0/3	0/3	-	-	0/3	0/3
**6g**	-CH_2_C_6_H_4_ (*o-*F)	3/3	0/3	1/3	0/3	0/3	0/3
**6h**	-CH_2_C_6_H_4_ (*m-*F)	0/3	0/3	-	-	0/3	0/3
**6i**	-CH_2_C_6_H_4_ (*p-*F)	1/3	0/3	-	-	0/3	0/3
**6j**	-CH_2_C_6_H_4_ (*o-*Cl)	1/3	0/3	-	-	0/3	0/3
**6k**	-CH_2_C_6_H_4_ (*m-*Cl)	0/3	0/3	-	-	0/3	0/3
**6l**	-CH_2_C_6_H_4_ (*p-*Cl)	0/3	0/3	-	-	0/3	0/3
**6m**	-CH_2_C_6_H_3 _(2,6-2Cl)	2/3	1/3	1/3	0/3	0/3	0/3
**6n**	-CH_2_C_6_H_4_ (*p*-CF3)	0/3	0/3	-	-	0/3	0/3
**6o**	-CH_2_C_6_H_4_ (*p*-CH3)	0/3	0/3	-	-	0/3	0/3
**blank**	-	0/3	0/3	0/3	0/3	0/3	0/3

^a^, Maximal electroshock: doses of 100 mg/kg or 300 mg/kg were administrated intraperitoneally in mice. The animals were examined at two times: 0.5 h and 4 h after administration; ^b^, Neurotoxicity test; ^c^, Not tests; ^d^ n1/n2, the animals protected/the animals tested.

**Table 2:** Quantitative Pharmacological Parameters ED_50_, TD_50_, and PI Values in Mice

Comp.	ED_50_^e^ MES (mg/kg)	TD_50_^f^ (mg/kg)	PI ^g^
6a	214.7 (135.4-340.4) ^h^	409.1 (357.9-467.6)	1.91
6b	190.9 (124.1-293.8)	435.2 (333.1-568.6)	2.28
6g	160.4 (113.0-227.6)	440.1 (372.9-519.4)	2.74
6m	214.4 (128.96-356.4)	432.1 (330.7-564.6)	2.02
VPA ^j^	216.9 (207.5-226.3)	372.9 (356.0-389.8)	1.72

^e^ ED50, median effective dose affording anticonvulsant protection in 50% of animals; ^f^ TD50, median toxic dose eliciting minimal neurological toxicity in 50% of animals; ^g^ PI, protective index (TD_50_/ED_50_); ^h^ 95% confidence intervals given in parentheses.

**Figure 2:**
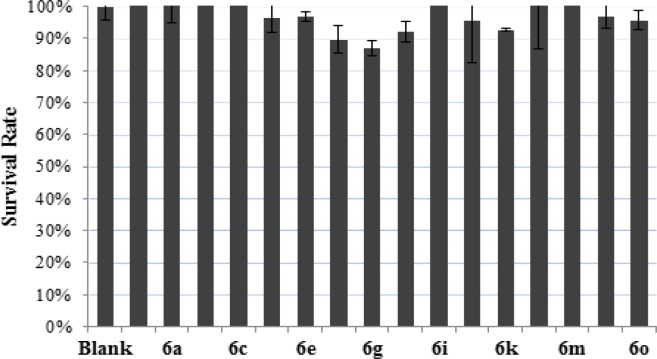
The Comparison of Cytotoxicity of the Compounds. The ordinate represents survival rate of the cell line, the abscissa is the synthesized compounds, and blank group was untreated by any compounds

**Figure 3:**
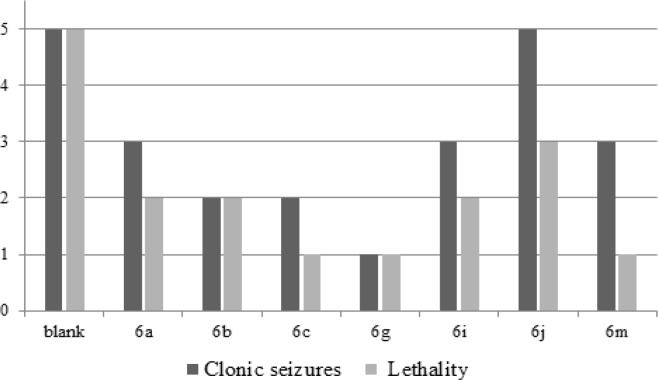
Effects of the Target Compounds (100 mg/kg) on s.c. Pentylenetetrazol -Induced Seizures in Mice. The ordinate represents the number of attacked mice with the clonic seizures or lethality on the test mice, and the abscissa is the synthesized compounds. The blank group was untreated by any compounds

*Pharmacology*

The anticonvulsant activity evaluation of all the compounds were determined using the MES test, which is a mechanism-independent animal seizure model that enables the identification of compounds preventing seizure spread (Rogawski, 2006). It should be noted that the MES model remains the most useful tool for the identification of new anticonvulsants, despite significant advances in epilepsy research in the past several years (Castel-Branco et al., 2009). The MES seizure model was used for preliminary screening of compounds **4, 5** and **6a-6o**. They were administered to mice intraperitoneally (i.p.) at the fixed dose of 100 mg/kg and the anticonvulsant protection was observed at two post-treatment times: 0.5 and 4 h. The method applied here allowed the determination of the number of animals (in a group consisting of three mice) protected against electrically induced seizures as well as the estimation of the time course of anticonvulsant activity including quick-acting (0.5 h) or long-acting properties (4 h). The results are presented in Table 1. The preliminary pharmacological screening revealed that four compounds (**6a, 6b, 6g** and **6m**) showed about 33% anticonvulsant protection in the 0.5 h period at the dose of 100 mg/kg, but none of them had activity in the 4 h period at the same dose. None of all the compounds presented neurotoxicity at the dose of 300 mg/kg. 

On the basis of the preliminary screening results, compounds **6a, 6b, 6g **and **6m** were subjected to the next phase of trials regarding the quantification of their anticonvulsant activity in mice. The results of the quantitative tests are reported in Table 2, along with the data from sodium valproate as the positive drug control group. As shown in Table 2, all of the three compounds showed similar or stronger anticonvulsant activities than sodium valproate (the ED_50_ was 216.9 mg/kg). Especially, compound **6g** showed better activities (the ED_50_ was 160.4 mg/kg) and higher safety (the PI was 2.74) than other compounds, including the positive drug.

*In vitro cytotoxicity*

All of the synthesized compounds (**6a-6o**) were evaluated for their cytotoxicity against cell lines to demonstrate the safety. Form Figure 2, we could see that the synthesized compounds showed little or no cytotoxicity to the cell line at 20μM. The survival rate of the cells treated with target compounds exceeded 85%.

*Chemical-induced seizure tests speculated on the mechanism*

The compounds, which showed anticonvulsant activities in the preliminary screening at 300 mg/kg, were infused to stomach of mice to observe the effect on the convulsion of the pentylenetetrazol model. As shown in Figure 3, the results were similar to the MES model, compounds **6b, 6c** and **6g** showed better activity against PTZ in varying degrees. Especially, compound **6g** (o-F) showed about 80% anticonvulsant protection in the 1.5 h period at the dose of 100 mg/kg in this group of five mice. And although the compounds **6a, 6j** and **6m** had weaker effective against scPTZ model, they were able to resist PTZ-induced lethality to some extent in mice.

## Discussion

Based on the activity screening results, the following structure-activity relationships (SAR) were obtained, while analyzing the preliminary screening of the synthesized compounds. Among the six alkyl chain-substituted derivatives, **6a** and **6b** showed better activities. And **6b **was also a little better than **6a**. However, with the increase in length, the activities of the compounds did not increase. Compound **6f**, substituted with a benzyloxy group at the 6-position of the benzothiazole core, showed no activity at 100 mg/kg. But when the F, Cl and so on groups were subsequently added onto the benzyloxy group, some compounds showed activities. Substituent position on the phenyl ring also influenced anticonvulsant activity in the 6-fluorobenzyl derivatives as *o*-F > *p*-F > *m*-F. However, o-Cl showed activity at the dose of 300 mg/kg and all the 6-chlorobenzyl derivatives showed no activity at the dose of 100 mg/kg. Only compound **6m**, with two added chlorine atoms, showed activity in the 0.5 h period. When the substituent was changed to the electro-donating substituent (**6o**), the anticonvulsant activity disappeared.

As we known that clinically antiepileptic drug normally had a narrow therapeutic index, it was important to evaluate the effects of synthesized compounds on cell lines. From the experimental results, it could be deduce that this series of compounds was also safety to the cell line. Of course, testing only that way is insufficient. More extensive and thorough toxicological studies would play an important role in the further study towards safety on these compounds at the next step.

The scPTZ model employs chemically (pentylenetetrazol) induced myoclonic seizures and allows the identification of agents raising the seizure threshold. This test is related to human generalized absence seizures (Löscher, 2011). The research shows, PTZ has been reported to produce seizures by inhibiting γ-aminobutyric acid (GABA) neurotransmission (Löscher et al., 2011; Okada et al., 1989). GABA is the main inhibitory neurotransmitter in the brain, and is widely implicated in epilepsy. Inhibition of GABAergic neurotransmission or activity has been shown to promote and facilitate seizures (Gale, 1992). 

But the effect target of the compounds was not clear enough and the specific molecular mechanism of inhibition of GABAergic neurotransmission remained unclear. Next steps, further study towards determining the mechanisms of action and effect target of these compounds is currently underway in our laboratory.

Although many underlying disease mechanisms can lead to epilepsy, the cause of the disease is still unknown in about 50% of cases globally. Up to 70% of people living with epilepsy could become seizure free with appropriate use of antiepileptic medicines. But in low-income countries, about three quarters of people with epilepsy may not receive the treatment they need. This is called the “treatment gap” and the average availability of generic antiepileptic medicines in the public sector of low- and middle-income countries to be less than 50%. This may act as a barrier to accessing treatment. Besides developing more inexpensive drugs, training primary health-care providers to diagnose and treat epilepsy can effectively reduce the epilepsy treatment gap. More effective projects should be carried out in many countries to reduce the treatment gap and morbidity of people with epilepsy, to train and educate health professionals, to dispel stigma, to identify potential prevention strategies, and to develop models integrating epilepsy care into local health systems.

Taken together, in the present study, we described the synthesis and anticonvulsant activity evaluation of 2-(3,5-dimethyl-1H-pyrazol-1-yl)-6-alkoxybenzo[d]thiazole (**6a-6e**) or 2-(3,5-dimethyl-1H-pyrazol-1-yl)-6-benzylbenzo[d]thiazole (**6f-6o**). All the compounds were tested for their anticonvulsant activity and neurotoxicity using the MES and rotarod tests at the dose of 100 mg/kg. Among them, compounds **6g** showed the most active in this study in the MES assay and least neurotoxicity, and that made **6g** had higher safety than marketed drugs sodium valproate. In addition, all the synthetic compounds, including **6g**, had shown little cytotoxicity in the MTT test. It was well to be reminded that compound **6g** demonstrated antagonistic activity against seizures induced by pentylenetetrazol. It suggested that compound 6g maybe exert anticonvulsant activity through effecting GABAergic neurotransmission in the brain. We hoped that this research established foundation to reveal the mechanism of antiepilepsy and anticonvulsant and to promote a treatment orientation.
